# Biogeography and Adaptive evolution of *Streptomyces* Strains from saline environments

**DOI:** 10.1038/srep32718

**Published:** 2016-09-06

**Authors:** Fei Zhao, Yu-Hua Qin, Xin Zheng, Hong-Wei Zhao, Dong-Yan Chai, Wei Li, Ming-Xiang Pu, Xing-Sheng Zuo, Wen Qian, Ping Ni, Yong Zhang, Han Mei, Song-Tao He

**Affiliations:** 1 Pharmaceutical deparment, Henan Province People’s Hospital, No.7, Wei Wu Road, Zhengzhou, Henan, 450003, China; 2 Yunnan WALVAX Biotechnology Co., Ltd, Kunming, 650106, China; 3 Yuxi WALVAX Biotechnology Co., Ltd, Kunming, 653100, China; 4 Eryuan No. one high school, Dali Bai nationality Prefecture, 671202, China; 5 Yunnan Weather Modification Center, Kunming, 650034, China; 6 Key Laboratory of Microbial Diversity in Southwest China, Ministry of Education, Yunnan Institute of Microbiology, Yunnan University, Kunming, 650091, China

## Abstract

The genus *Streptomyces* is a widespread genus within the phylum Actinobacteria and has been isolated from various environments worldwide. However, little is known about whether biogeography affects distributional pattern of *Streptomyces* in salty environments. Such information is essential for understanding the ecology of *Streptomyces*. Here we analyzed four house-keeping genes (16S rRNA, *rpoB*, *recA* and *atpD*) and salty-tolerance related genes (*ectA*-*ectD*) of 38 *Streptomyces* strains isolated from saline environments in Yunnan and Xinjiang Provinces of western China. The obtained *Streptomyces* strains were classified into three operational taxonomic units, each comprising habitat-specific geno- and ecotype STs. In combination with expressional variations of salty-tolerance related genes, the statistical analyses showed that spatial distance and environmental factors substantially influenced *Streptomyces* distribution in saline environments: the former had stronger influence at large spatial scales (>700 km), whereas the latter was influential at large (>700 km) and small spatial scales (<700 km). Plus, the quantitative analyses of salty-tolerence related genes (*ectA*-*D*) indicated that *Streptomyces* strains from salt lakes have higher expression of *ectA*-*D* genes and could accumulate larger quantities of ectoine and hydroxyectoine than strains from salt mines, which could help them resist to salinity in the hypersaline environments.

The study of biogeography addresses spatial patterns of species and ecosystems in geographic space or through geological time^1^. It is widely accepted that macro-organism populations have many similar characteristics and common ancestry and thus possess different geographical distributions due to geographical isolation^2^. In contrast, debate has been lasting on microbial biogeography in that microorganisms have great potential for global dispersal and adaptability to diverse environments^3^. However, numerous studies recently reported biogeographic patterns for microbial distributions^4^,^5^.

Microbial biogeography is mainly ascribed to two factors: the environmental heterogeneity and spatial distance, either of which alone cannot entirely account for geographic distribution patterns of some widespread bacterial groups^6^,^7^. However, little is known about the relative importance of these two factors to shaping microbial biogeography in saline and saline environments. Theoretically, saline environments are suitable for studying impact of environmental effects on bacterial biogeography because of strong environmental selective pressures in such environments^8^,^9^. Previous studies have shown that spatial distance and environmental factors contribute differently to shaping microbial biogeography in saline environments with respect to certain microbial groups^9^,^10^.

As one group of widespread Actinobacteria, *Streptomyces* species have drawn extensive attention from microbiologists due to their capacity to produce diverse bioactive compounds such as antibiotics^11^,^12^. Many characterized *Streptomyces* strains were largely recovered from various salty habitats, such as marine environments, deserts, alkaline or saline soils^13^,^14^. In these salty environments, *Streptomyces* spp. underwent a wide range of environmental pressures and thus developed distinct genetic and metabolic features among different habitats^15^. So in order to understand the underlying reasons for the endemicity of *Streptomyces* species in various saline environments, it is important to know 1) whether geographic isolation affects distributional pattern of *Streptomyces* in saline environments and 2) relative importance of spatial distance and environmental factors to shaping *Streptomyces* distribution in saline environments.

Investigation on biogeography of *Streptomyces* requires a detailed taxonomic classification of *Streptomyces* strains from different saline habitats (salt mines vs lakes). High phylogeny resolution can be achieved by multilocus sequence analyses, which combine phylogenies of 16S rRNA and functional housekeeping genes such as *rpoB*, *recA* and *atpD*^16^. Previous studies have shown that 16S rRNA gene together with *rpoB*, *recA* and *atpD* genes could provide better phylogenetic resolution than one single gene^16^. Furthermore, the expressional variation of salty-tolerence related genes (*ectA-ectD*) could show adaptive evolution of *Streptomyces* strains in saline environments^17^. Here, we applied a multilocus phylogenetic analysis of four house-keeping genes (16S rRNA, *rpoB*, *recA* and *atpD*) and salty-tolerence related genes (*ectA-ectD*) to investigate the biogeographic patterns of *Streptomyces* strains retrieved from the saline environments in Xinjiang and Yunnan Provinces of western China, and assessed relative effects of spatial distance and environmental factors on biogeography and salty-tolerence mechanism of *Streptomyces* strains from different salty-habitats (salt mines vs lakes) by Q-PCR analyses.

## Results

### Geochemistry differentiations between two salty-habitat types (salt mines vs lakes)

**Figure 1:**
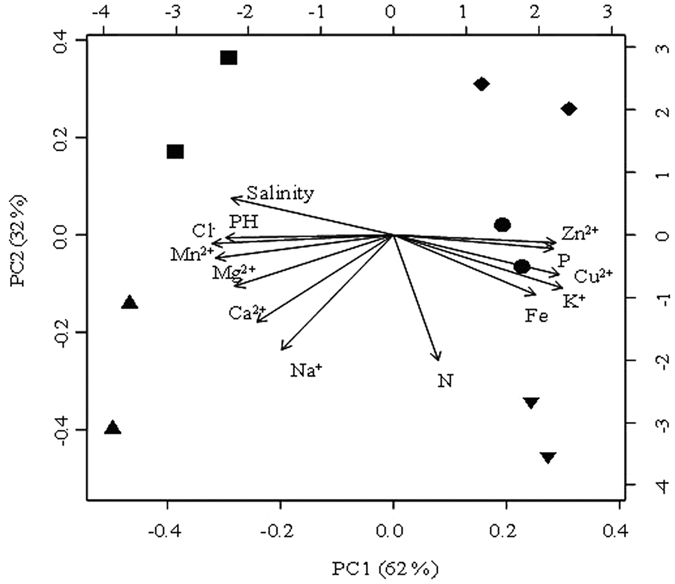
A PCA map showing the 10 sampling sites and their correlation with trace elements. Note: pH, Cl^−^, Ca^2+^, Mg^2^, K^+^, Na^+^, Fe^2^, Mn^2^, Cu^2^, Zn^2^, Sanlinity, total N (nitrogen) and total P (phosphorus) were used to evaluate the influence of each variable. The longer the arrow, the greater the influence; the smaller the angle between two arrows, the closer their correlation. Solid squares (■) and pright triangles (▲) denote Qijiaojing (QJJ) and Aydingkol (AK) salt lake sites in Xinjiang Province; inverse triangles (▼), diamonds (♦) and black circles (•) denote Jiangcheng (JC), Heijing (HJ), Yunlong(YL) salt mine sites in Yunnan Province, respectively.

In Yunnan Province, Heijing, Yunlong and Jiangcheng salt mines are under subtropical monsoon climate, and originated from salty beds or basins formed by ancient lakes more than 120,000 years ago^18^. The salt ores in the salt mines are rich in sodium chloride as well as potassium chloride. In Xinjiang Province, the Aydingkol and Qijiaojing salt lake were formed from Holocene of the late Quaternary^18^. As a result of strong evaporation more than 1000 years, most of surface areas of Aydingkol and Qijiaojing salt lakes have been highly mineralized, containing abundant alkaline rock salt (e.g., Glauber’s salt, glauberite, gypsum, sodium chloride)^18^, but low concentration of potassium salt. Principle component analysis (PCA) showed that the Yunnan sampling sites were different from that of Xinjiang with respect to environmental factors: the sediment chemistry of the Aydingkol and Qijiaojing salt lakes was different from that of the salt mines in Yunnan Province: the former possesses higher salinity, pH and concentrations of Ca^2+^, Mg^2+^ and Mn^2+^ but lower concentrations of trace elements (e.g. K^+^ and Zn^2+^) and total phosphorus than the latter (cumulative contribution value = 94%, Fig. 1). In addition, the sampling sites within a salty-habitat type (salt mines or lakes) were different from each other on the basis of climate types, geographic distances, and geochemistry factors (Tables S1 and S2, Fig. 1).

### Phylogenetic analysis of four house-keeping genes (16S rRNA, *rpoB*, *recA* and *atpD*) and salty-tolerence related genes (*ectA-ectD*)

**Figure 2:**
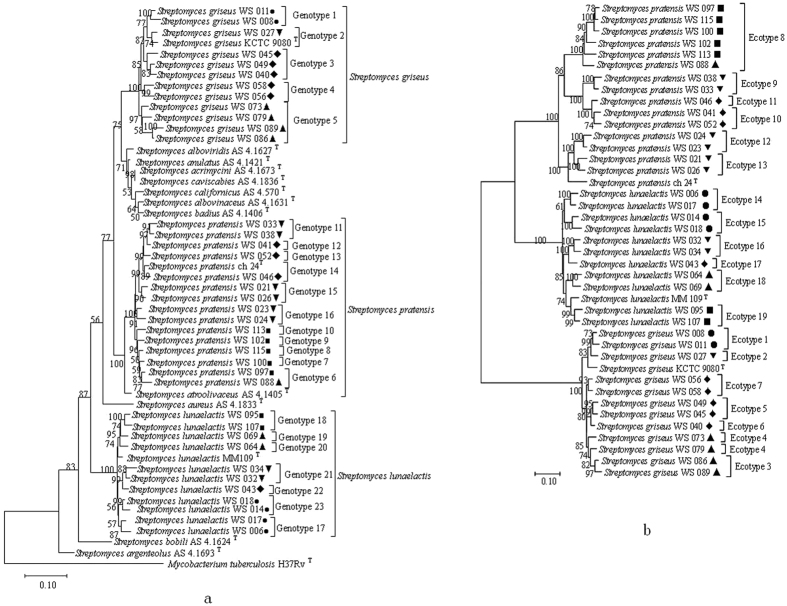
Maximum Likelihood based Phylogenetic trees of concatenated sequences of four house-keeping genes (indicated by A, 16S rRNA, *rpoB*, *recA* and *atpD*) and salty-tolerented related genes (indicated by B, ectA-D), showing endemism for three *Streptomyces* OTUs. Bar, 0.1, ten nucleotide substitutions per 1000 nt; Bootstrap values are shown as percentage of 1000 replicates, and only the bootstrap values above 50% are shown. Solid squares (■) and pright triangles (▲) denote Qijiaojing (QJJ) and Aydingkol (AK) salt lake sites in Xinjiang Province; inverse triangles (▼), diamonds (♦) and black circles (•) denote Jiangcheng (JC), Heijing (HJ), Yunlong(YL) salt mine sites in Yunnan Province, respectively. The geno- and ecotype STs are marked in the clades, and each genotype is supported by high bootstrap value (>80%).

A total of 38 *Streptomyces* strains were retrieved and subjected to phenotypic characterization^14^ as well as OTU identification. The obtained *Streptomyces* strains belonged to three OTUs (*Streptomyces pratensis*, *Streptomyces griseus*, and *Streptomyces lunaelactis*) (Table S3). Each of the identified OTUs covered more than eight strains and contained at least one strain from a sampling site (Table S3). The multi-locus sequence typing (MLST) phylogeny showed endemism of *Streptomyces* strains: each endemic geno- and ecotype ST was specific to a site or one salty-habitat type (salt mines or lakes, Table 1 and Fig. 2 and Fig. S1, Bootstrap value >80%). A total of 23 genotype (Fig. 2A and Fig. S1A) and 19 ecotype STs (Fig. 2B and Fig. S1B) were identified, with either salty habitat type containing at least 10 geno- or ecotype STs (Fig. 2 and Fig. S1, Table 1), and each sampling site including at least 3 geno- or ecotype STs (Table 1 and Table S5).

### Effects of spatial distance and environmental factors on the geographic patterns of *Streptomyces* strains

**Figure 3:**
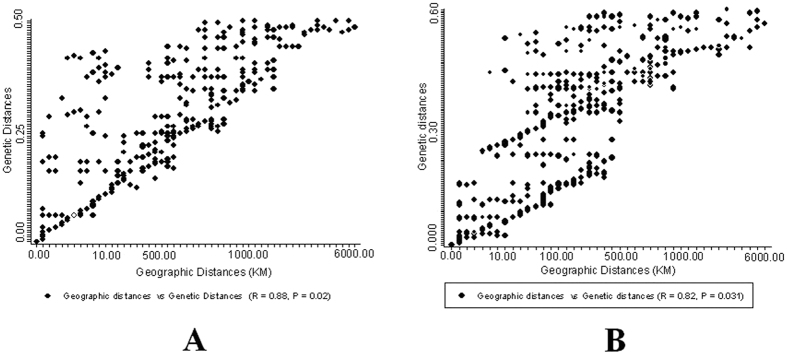
Mantel correlation between genetic distances of the investigated genes and geographic distance for the three identified *Streptomyces* OTUs. (**A,B**) panels indicate the correlation between geographic distance and genetic distances of concatenated sequences of four house-keeping genes (16S rRNA, *rpoB*, *recA* and *atpD*, r = 0.88, p = 0.02) and salty-tolerented related genes (ectA-D, r = 0.82, p = 0.031), respectively.

MLST of four housekeeping genes and *ectA*-*D* revealed significant correlations of genetic divergence with geographic distance (MLST of four house-keeping genes, R = 0.88, p = 0.02; MLST of four salty-tolerence related genes, R = 0.82, p = 0.031) (Fig. 3). In addition, the closely related geno- or ecotype STs were present within a very small scale (geographic distances within salt mine or saline lake habitat <700 km, the genetic divergences <0.10; and geographic distances within a sampling site <100 km, the genetic divergences <0.05) but not at distant locations (spatial distances between salt mines and lakes >700 km, the genetic divergences >0.10) (Fig. 3). Mantel test (r > 0.5, P < 0.05) showed that the differentiations of ecotype STs were significantly correlated with the geochemistry variations of sediments between Yunnan salt mines and Xinjiang salt lakes (such as, Na^+^, Mg^2+^, Cl^−^ and salinity, Table 2).

The Z-test of four salty-tolerence related genes (*ectA*-*D*) by optimum ‘positive selection’ models (M2a and M8) showed that evolutions of these genes in the 38 *Streptomyces* strains in this study were partially under positive environmental selection (mean dN/dS > 1, respectively, Table 3 and Fig. S3), while the “nearly neutral” model (Mla) indicated that three house-keeping genes (*rpoB*, rec*A*, and *atpD*, mean dN/dS < 1, Table 3) experienced a neutral evolution in three *Streptomyces* OTUs.

### Variation of *Streptomyces* in response to abiotic stress at 5%, 10% and 15% NaCl

**Figure 4:**
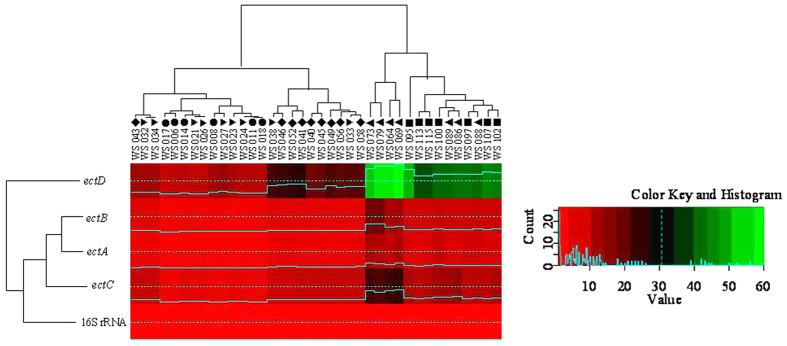
Relative expression quantitation of *ectA*-*D* genes at 15% of NaCl by Q-PCR. Solid squares(■), pright triangles (▲), inverse triangles (▼), diamonds (♦) and black circles (•) indicate *Streptomyces* strains from Qijiaojing (QJJ) and Aydingkol (AK) salt lake sites in Xinjiang Province and Jiangcheng (JC), Heijing (HJ), Yunlong(YL) salt mine sites in Yunnan Province, respectively. Each deviation of fold changes was calculated by comparison expression of *ectA*-*D* in *Streptomyces* cells growing at salt treated ISP3 medium (5% and 15% g/ml of NaCl) with normal (0% g/ml of NaCl) medium.

**Figure 5:**
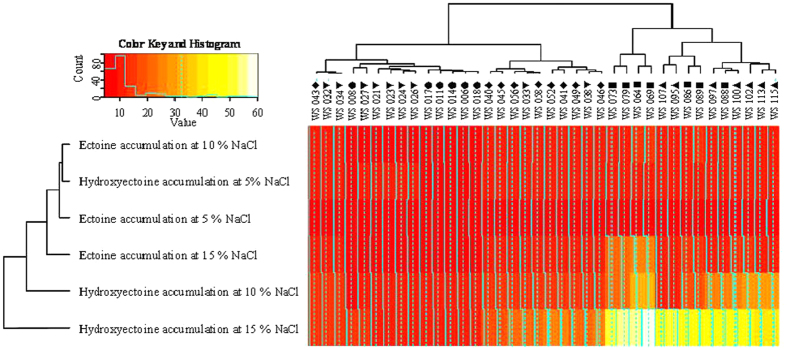
Relative quantitation of accumulations of ectoine and hydroxyectoine at different concentrations of NaCl by HPLC. Solid squares (■), pright triangles (▲), inverse triangles (▼), diamonds (♦) and black circles (•) indicate *Streptomyces* strains from Qijiaojing (QJJ) and Aydingkol (AK) salt lake sites in Xinjiang Province and Jiangcheng (JC), Heijing (HJ), Yunlong(YL) salt mine sites in Yunnan Province, respectively. For estimating fold changes in accumulation of ectoine and hydroxyectoine, 1 fold changes was defined as the accumulation of ectoine and hydroxyectoine in *Streptomyces* cells that were grown at the ISP3 medium without salt (0% g/ml of NaCl), each deviation of fold changes was calculated by comparing quantitation of ectoine and hydroxyectoine in *Streptomyces* cells growing at salt treated ISP3 medium (5% and 15% g/ml of NaCl) with normal (0% g/ml of NaCl) medium.

With the help of Q-PCR and HPLC, responses of 38 *Streptomyces* strains to high osmotic stress (up to 5% NaCl) were investigated by means of quantitative expression of *ectA*-*D* genes and quantitation of biosynthesis of ectoine and hydroxyectoine. As a result, the expressional variation of *ectA*-*D* (especially, *ectC*-*D*) revealed strains from Xinjiang salt lakes have higher expression of *ectA*-*D* (especially, *ectC *> 20 fold changes; *ectD *> 30 fold changes, Fig. 4) and could accumulate larger quantities of ectoine (>10 fold changes, Fig. 5) and hydroxyectoine (>15 fold changes, Fig. 5) in response to high osmotic stress than strains from salt mines in Yunnan at 15% g/ml of NaCl (Fig. 4, *ectC* > 8 fold changes; *ectD* > 10 fold changes; Fig. 5, ectoine >5 fold changes and hydroxyectoine >7 fold changes).

## Discussion

### Geographic patterns and endemism of *Streptomyces* geno- and ecotype STs within an OTU

Our study supports previously detected biogeographical patterns among *Streptomyces* from saline environments, being consistent with the fact that some *Streptomyces* species have been exclusively isolated from certain habitats to date^13^,^14^,^19^. The patchy distribution of *Streptomyces* among species and endemic patterns within one species (Fig. 2 and Fig. S1) indicated that biogeography may influence microbial distribution within a species but may not function among species within the genus at a large geographic scale^1^. The observed endemic distribution of *Streptomyces* strains was consistent with previous studies about other microbial groups^1^,^8^,^9^. For example, a patchy geographic distribution was found for the bacterial isolates within a homogeneous background (sulfate-reducing sediments from four continents^20^. Similarly, crenarchaeal assemblages in mesophilic soil habitats were distributed in mosaic patterns of different phylotypes^21^. Likewise, individual genotypes of purple non-sulfur bacterium Rhodopseudomonas palustris and *Nocardiopsis* were detected only locally and exhibited patchy distribution at 1 km or 100 km scales^9^,^22^.

### Relative importance of spatial distance and environmental factors upon endemism of *Streptomyces* strains

The biogeographic distribution of *Streptomyces* could be ascribed to spatial distance and environmental factors^1^,^6^. However, little is known about the relative importance of spatial distance and environmental factors on the distributional patterns of the three *Streptomyces* species. In this study, the impact of spatial distance upon endemicity of *Streptomyces* from salt mines in Yunnan or salt lakes in Xinjiang could be validated by the fact that the closely related geno- or ecotype STs was present within a very small scale of local habitats (geographic distances between salty mines ot lakes <700 km) but not at distant (spatial distances between salty mines and lakes >700 km) habitats (r > 0.80, p < 0.005, Fig. 3). This indicated that spatial distance significantly contribute to the observed biogeographic patterns of *Streptomyces* strains from different salty habitats, which was consistent with some previous studies^1^,^6^,^23^,^24^. Previously, spatial distance together with genetic drift or physical isolation was proposed to lead to endemism of microbial populations at a continent scale or from different biotopes^24^.

Our data suggest that the spatial distance notably resulted in differentiations of *Streptomyces* strains between two salty-habitat types (spatial distances between salty mines and lakes >700 km, the genetic divergences >0.10; R > 0.80, P < 0.05, Fig. 3). Our study suggested that both environmental parameters and spatial distance played a role in biogeography of *Streptomyces* strains at the large (between salt mines and salt lakes) scale. However, environmental parameters apparently rather influence microbial endemism local (within a salty-habitat type) scale. In the present study, the genetic differentiations of four salty-tolerence related genes of retrieved *Streptomyces* strains significantly corresponded to heterogeneities of some cations or anions in the sediments of the studied sampling sites between salt mines and lakes, or even some sampling sites within a salty-habitat type (Na^+^, Cl^−^, Mg^2+^ and salinity, Table 2). This observation was consistent with some previous studies, in which environmental factors rather than spatial distance were shown to cause bacterial variation at a local scale (from 1 km to 100 km)^25^. Previous studies indicated that Na^+^, Mg^2+^, and Cl^−^ were significant in influencing bacterial biogeography at the species (97% OTU) or subspecies (99%) levels^26^,^27^. The Na^+^ ions were important to some halophilic bacteria or alkaliphilic bacteria as they replaced protons and coupled ion to cope with the high external pH, rather than increasing the electric potential difference across the cytoplasmic membrane^26^. Mg^2+^ was a chaotropic agent and a limiting factor in the diversity of microbes in the saline environment^27^. In addition, salinity of sediments was important regulators of some extremozymes in *Streptomyces* genus, for example, xylanases, alpha amylases, thermoalklotolerant *β*-1,3-glucanases and cellulases^17^, which is also consistent with our study in that *Streptomyces* strains from Xinjiang salt lakes have higher abilities of regulating expression of *ectA*-*D* genes (especially, *ectC* > 20 fold changes; *ectD *> 30 fold changes, Fig. 4) and biosynthetic potential of larger quantities of (>10 fold changes, Fig. 5) and hydroxyectoine (>15 fold changes, Fig. 5) than strains from Yunnan salt mines at salty-treated ISP3 medium (Fig. 4, *ectC *> only 8 fold changes and *ectD *> only 10 fold changes; Fig. 5, ectoine >5 fold changes and hydroxyectoine >7 fold changes). Thus, it is reasonable to observe the significant influence of environmental factors on endemism of *Streptomyces* strains. Environmental factors influenced some functional genes important for bacterial survival more significantly than house-keeping genes. For example, evolution of *ectA-D* genes of the obtained *Streptomyces* strains showed more visible selection within one habitat or one region than the highly conserved house-keeping genes (*rpoB*, *recA* and *atpD*, Table 3 and Fig. S3). While *ectB* and *ectD* gene is responsible for biosythesis of hydroxyectoine that could help *Streptomyces* strains to deal with external osmotic pressure *in vitro*. Some residues in catalytic regulation domains of *ectB* and *ectD* is under positve selection and subjected to resistence to highly salinity from hypersaline environments (Fig. S3). The abiotic pressure (e.g. salinity) could result in comparably more rapid evolution of *Streptomyces* strains in the *ectB* and *ectD* gene than the *ectA* and *C* genes (Table 3 and Fig. S3; p value < = 0.05). This observation could be ascribed to the fact that some residues of the four salty-tolerence related genes could be subjected to mutation due to some abiotic stress (eg. salinity) (Table 3 and Fig. S3; p value < = 0.05), which leads to the catalytic regulation function of their biosynthesis of hydroxyectoine^17^.

In summary, *Streptomyces* spp. in saline environments possessed geographic distribution patterns between salt mines and lakes. Spatial distance and environmental factors influenced the biogeography distribution of *Streptomyces* from different slaty-habitats at large (>700 km) and local scales (<700 km), respectively. Furthermore, environmental factors (e.g. salinity) could result in comparably more rapid evolution of *Streptomyces* strains in some salty-tolerence related genes than highly conserved house-keeping genes.

## Material and Methods

### Site description and sample collection

In this study, two sites each were sampled at the Heijing saline mine (HJ1, an abandoned salt mine; HJ2, a natural saline spring), Yunlong (YL1, an abandoned salt spring; YL2, a natural saline mine) and the Jiangcheng salt mine (JC1, an abandoned salt mine; JC2 site, a natural saline spring), respectively. Two (AK1 and AK2) and two sites (QJJ1 and QJJ2) were sampled at Aydingkol and Qijiaojing salt lakes, respectively. At each selected sampling site, sediments were sampled at the 10–30 cm depth and collected into sterile 50 ml sterile Falcon centrifuge tubes. GPS coordinates were recorded at each sampling point with a portable meter in the field and were subsequently imported into Map-Source according to the manufacturer’s instructions to measure the geographic distances among the sites. The location map of sampling sites was generated by Google earth 7.1 in the study. Qijiaojing Salt lake is more than 126 km away from Aydingkol Salt Lake in Xinjiang Province; while in Yunnan Province, Heijing Salt Mine is about 560 km away from Jiangcheng Salt Mine, and Yunlong Salt mines is about about 730 Km and 640 Km away from Heijiang and Jiangcheng salt mines. The sampling sites of Xinjiang Province are about 4300 km away from those of Yunnan Province (Table 1 and S1). The samples for microbial cultivation and geochemistry measurement were stored at 4 °C in the field and during transportation.

### Geochemistry measurements

The pH and salinity of 10 g sampled sediments were measured with portable meters after sediments being dissolved into 100 mL distilled water. The concentrations of major cations and trace elements in sediments from ten sampling sites were measured by flame atomic absorption spectrometry (HITACHI Z-2310). Total nitrogen of the sediment samples was determined by the semi-micro-Kjeldahl method and total phosphorus of the sediment samples was determined by the alkali fusion–Mo-Sb Anti-spectrophotometric method^28^. Principle component analysis (PCA) of the studied sediment samples was performed with the use of the R program^29^.

### Isolation of *Streptomyces* strains

The sediment samples (2 g, wet weight) were dispersed into 18 ml sterilized physiological saline water (con. 0.70%, w/v, equal to bacterial cell physiological salinity) and were incubated at 30 °C for 30 min with shaking at 150 rpm. The resulting slurry was serially diluted with sterilized physiological saline water (NaCl con. 0.70%, w/v). Aliquots (0.2 ml) of each dilution were spread onto petri dishes containing three different media: cellulose-casein multi-salt medium and modified ISP 2 and ISP 3 media^8^. All the agar plates were supplemented with 5% (w/v) NaCl and potassium dichromate (15 mg/L). The petri dishes were incubated at 37 °C for 4–6 weeks to obtain enough colonies of *Actinobacteria* strains. Based on the morphologic characteristics of *Streptomyces* spp. described previously^14^, some branching myceliums in petri dishes were picked and checked by light microscopy (BH-2; Olympus). Candidate strains were purified on inorganic salts-starch agar supplemented with 2% (w/v) NaCl and cultivated using the ISP3 medium (Difco Laboratories, Detroit, Mich) at 37 °C for four weeks. Genomic DNA of the obtained strains was extracted and 16S rRNA genes were PCR amplified^16^. PCR amplification of *rpoB*, *recA*, *atpD* and *ectA*-*D* genes was performed according to the methods described previously^16^,^17^. The amplified PCR products were purified using a TaKaRa DNA fragment purification kit (Ver. 2.0) and were sequenced using an ABI 3100 automated sequencer with primers of four genes (16S rRNA, 27f and 1525r; *rpoB*, *rpoBPF* and *rpoBPR*; *recA*, *recAF* and *recAR*; *atpD*, *atpDF* and *atpDR*; *ectA*, *ectAF* and *ectAR*; *ectB*, *ectBF* and *ectBR*; *ectC*, *ectCF* and *ectCR*; *ectD*, *ectDF* and *ectDR*) at Shanghai Sangon Biotech (Shanghai, China)^16^,^17^. The 16S rRNA gene sequences obtained from the candidate strains were compared with reference taxa via the EzTaxon-e database. The sequences similarity levels were calculated between the candidate strains and their related *Streptomyces* taxa in the EzTaxon-e database^30^.

### Phylogenetic analysis of isolated *Streptomyces* strains

Multiple alignments and genetic distance calculations were carried out by using CLUSTAL_X53^31^ after retrieving the reference sequences of *Streptomyces* type strains from the EzTaxon-e database. The pair-wise similarities between *Streptomyces* strains were calculated by the software package MEGA 4.0^32^. OTU classification was performed using DOTUR appliying a 97% 16S rRNA sequence similarity cut-off. The 97% identity of 16S rRNA gene sequences corresponded to 70% of DNA-DNA relatedness, which was widely used as the cutoff value for species definition in prokaryotes^33^. Reference sequences were retrieved from NCBI (National Center for Biotechnology Informatics, http://www.ncbi.nlm.nih.gov) with BLAST (Basic Local Alignment Search Tool, http://blast.ncbi.nlm.nih.gov/Blast.cgi). Subsequently, in order to study the biogeographic pattern of *Streptomyces*, four house-keeping (16S rRNA, *rpoB recA* and *atpD*) were assigned with allele numbers according to the multi-locus sequence typing (MLST) web site (www.mlst.net)^16^. And a multi-locus sequence type (ST) of concatenated sequences of four house-keeping genes within an OTU was defined as a genotype ST, and a ST of concatenated sequences of *ectA*-*D* within an OTU was nominated as one ecotype ST according to their correlation with multi-locus sequence type of four house-keeping genes (16S rRNA, *rpoB*, *recA* and *atpD*). Phylogenies of the concatenated sequences of investigated genes were constructed with Bayesian inference by using the PhyML 1.8.3 software with maximum-likelihood method and Mr Bayes-3.1.2^34^,^35^. Bootstrap analysis was used to evaluate the stability of tree topology by resampling 1000 times^34^,^36^,^37^.

### Biostatistic and bioinformatic analyses on the biogeographic patterns of *Streptomyces* strains

In order to assess the impact of spatial distances on *Streptomyces* strains’ dispersal, the correlations between Nei’s unbiased genetic distances of concatenated sequences of the investigated genes (16S rRNA, *rpoB*, *recA*, and *atpD*; *ectA-D*) and their corresponding geographic distances were analyzed using Mantel tests implemented in the NTSYS package^32^. Additionally, the relationship between differentiation in sediments geochemistry and variations of endemic geno- and ecotype STs among ten sampling sites were analyzed by simple Mantel test with the R program^29^. The maximum-likelihood method of Yang, implemented in the codeml program from the PAML package, was applied to analyze the effects of environmental forces on adaptive evolution of *Streptomyces* strains^38^. Six models were used to detect positive environmental selection upon evolution of the *Streptomyces* strains. Each model allows for various dN/dS ratios ω among sites, including the simplest model (M0 or one-ratio model), the ‘nearly neutral’ model (Mla), the positive selection model (M2a), the discrete Model M3, Model M7 (*β*), and the optimum positively selective Model M8.

### Estimating expression quatitation of *ectA*-*D* by Q-PCR and analysis of accumulations of ectoine and hydroxyectoine by High performance liquid chromatography (HPLC)

Total RNAs of the each growth condition was extracted from the cells grown in ISP3 medium using Trizol reagent (Invitrogen). One microgram of each RNA sample was used for constructing cDNA after it was treated with RNase-free DNase I (Invitrogen) using a cDNA Synthesis kit (BioRad). Q-PCR amplification of *ectA*-*D* and 16S rRNA (internal control) genes was performed according to the methods described previously^17^. Results were expressed as the normalized ratio of mRNA level of genes of interest (*ectA*-*D*) over internal control (16S rRNA gene). R program was employed to estimate the fold changes in expression of *ectA*-*D* genes and accumulation of ectoine and hydroxyectoine at 0%, 5% and 15% g/ml of NaCl via heatmap2 package^29^, each deviation of fold changes was calculated by comparison expression of *ectA*-*D* in *Streptomyces* cells growing at salt treated ISP3 medium (5% and 15% g/ml of NaCl) with normal (0% g/ml of NaCl) medium. And High performance liquid chromatography (HPLC) was employed to evaluate the accumulation of ectoine and hydroxyectoine in *Streptomyces* cells in response to osmotic stress according to the methods described previously^17^. For estimating fold changes in accumulation of ectoine and hydroxyectoine, 1 fold changes was defined as the accumulation of ectoine and hydroxyectoine in *Streptomyces* cells that were grown at the ISP3 medium without salt (0% g/ml of NaCl), each deviation of fold changes was calculated by comparing quantitation of ectoine and hydroxyectoine in *Streptomyces* cells growing at salt treated ISP3 medium (5% and 15% g/ml of NaCl) with normal (0% g/ml of NaCl) medium.

## Additional Information

**How to cite this article**: Zhao, F. *et al*. Biogeography and Adaptive evolution of *Streptomyces* Strains from saline environments. *Sci. Rep.* **6**, 32718; doi: 10.1038/srep32718 (2016).

## Supplementary Material

Supplementary Information

## Figures and Tables

**Table 1:** Geographic information for sampling sites and numbers of geno- and ecotype STs specific to a sampling site, a habitat, and a region.

Sampling Sites	Habitat types	Latitude (N) and Longitude (E)	Genotypes (Bootstrap values >80%)	Ecotypes (Bootstrap values >80%)
Yunnan Province	Salt mines		15	17
YL1		26° 21′N, 99° 4′E	2	2
YL2		25° 28′N, 98° 52′E	3	3
JC1		22° 35.069′N, 101° 50.034′E	2	2
JC2		22° 36.069′N, 101° 52.034′E	2	2
HJ1		25° 22.366′N, 101° 44.568′E	4	5
HJ2		25° 23.567′N, 101° 45.105′E	2	2
Xinjiang Province	Salt lakes		11	8
AK1		42° 29′N, 89° 22′E	3	2
AK2		42° 29′N, 89° 23′E	2	2
QJJ1		43° 26′N, 91° 38′E	2	2
QJJ2		43° 26′N, 91° 38.6′E	5	2

QJJ denotes Qijiaojing sampling sites, AK denotes Aydingkol sampling sites, YL denotes Yunlong sampling sites, JC denotes Jiangcheng sampling sites, and HJ denotes Heijing sampling sites.

**Table 2:** Simple Mantel test for geno- and ecotype STs of three *Streptomyces* OTUs in this study.

environmental factor	Genotype STs		ecotype STs	
	*r*^*2*^	p	*r*^*2*^	p
Cl^-^ (ppm)	0.436	0.066	**0**.**694**	**0**.**037**
Ca^2+^(ppm)	0.336	0.026	0.694	0.067
Mg^2+^(ppm)	0.336	0.028	**0**.**54**	**0**.**032**
Na^+^(ppm)	0.239	0.033	**0**.**706**	**0**.**046**
Fe^2/3+^(ppm)	0.431	0.054	0.497	0.055
Mn^2+^(ppm)	0.089	0.334	0.545	0.075
Cu^2+^(ppm)	0.201	0.043	0.59	0.048
K^+^(ppm)	0.405	0.052	0.433	0.045
Zn^2+^(ppm)	0.245	0.113	0.322	0.051
PH	0.602	0.201	0.05	0.04
Salinity (%)	0.662	0.078	**0**.**671**	**0**.**032**
Total N	0.04	0.055	0.452	0.033
Total P	0.032	0.056	0.055	0.033

*r*^*2*^ is the correlation value; positive or negative values reflect the type of relationship between the two matrices, while p is the probability associated with *r*^*2*^. *P* values are significant if *P* is <0.05 (boldface).

**Table 3:** Evaluation of positive evolution of seven genes of the studied *Streptomyces* strains by Z-test.

Gene	Model	Mean dN/dS (ω)	Log(L)	p-value
*rpoB*	M0 & Mla	0.378804	−2142.36	0.058000000
*recA*	M0 & Mla	0.494259	−1888.93	0.048843500
*atpD*	M0 & Mla	0.507579000	−2083.89	0.048527700
*ect A*	M2a & M8	**1**.**423620000**	−4847.17	0.035862800
*ect B*	M2a & M8	**1**.**638589000**	−10855.1	0.043130400
*ect C*	M2a & M8	**1**.**266699000**	−4212.67	0.048411300
*ect D*	M2a & M8	**1**.**526390000**	−5989.45	0.052474300

Two optimum positive selection models (M2a and M8) show presence of sites of the studied genes under positive selection, while model M0 & Mla indecated the studied genes experience a neutral evolution. The dN/dS ratios ω for each specific model were used to detect the studied genes undergoing positive selection, ω > 1 indicates residues of genes under positive selection. The *P* value for each specific model was employed to estimate significance of positive selections among seven genes.
