# Supplementary material for: Biogeography and Adaptive evolution of Streptomyces Strains from saline environments
Source: Sci Rep. 2016 Sep 6;6:32718. doi: 10.1038/srep32718 (PMC5011734; doi:10.1038/srep32718)
Supplement: Supplementary Information [file srep32718-s1.doc]

Biogeography and Adaptive evolution of *Streptomyces* Strains from saline environments

Fei Zhao^1^, Yu-Hua Qin^1^, Xin Zheng^2^, Hong-Wei Zhao^1^, Dong-Yan Chai^1^, Wei Li^3^, Ming-Xiang Pu^2^, Xing-Sheng Zuo^1^,Wen Qian^2^, Ping Ni^2^, Yong Zhang^5^, Han Mei^6^, Song-Tao He^1,2,4*^.

^1^Pharmaceutical deparment, Henan Province People’s Hospital, No.7, Wei Wu Road, Zhengzhou, Henan, 650003, China.

^2^Yunnan WALVAX Biotechnology Co., Ltd, Kunming, 650106, China.

^3^Yuxi WALVAX Biotechnology Co., Ltd, Kunming, 653100, China.

^4^Key Laboratory of Microbial Diversity in Southwest China, Ministry of Education, Yunnan Institute of Microbiology, Yunnan University, Kunming, 650091, China

^5^.Eryuan No. one high school, Dali Bai nationality Prefecture, 671202, China

^6^Yunnan Weather Modification Center, Kunming, 650034, China

**Correspondence:**

**Song-Tao He:** [walvaxtc@hotmail.com](mailto:walvaxtc@hotmail.com), [hesongtao86@hotmail.com](mailto:hesongtao86@hotmail.com), (86)-18687758000

**Author contributions**

F.Z., and S.H. conceived and designed the work; Y.Q., X.Z., and H.Z. performed the experiments; W.L., X.P. and X.Z. retrieved the sequences; D.C., H.M., W.Q., P.N. and Y.Z. analyzed the data. All of the authors assisted in writing the manuscript, discussed the results and commented on the manuscript.

**Additional information**Competing financial interests: The authors declare no competing financial interests.

**Table S1. Major ions and trace elements of sediment samples among 10 sampling sites**. QJJ and AK indicate the Qijiaojing and Aydingkol sampling sites in Xinjiang Province, respectively; and JC and HJ indicate the Jiangcheng and Heijing sampling sites in Yunnan province, respectively. Abbreviation of the sample names in the following tables and figures are same as Table S1 unless specified otherwise.

| **Major ions and trace elements of sediment samples** | **Sampling sites** | | | | | | | | | |
| --- | --- | --- | --- | --- | --- | --- | --- | --- | --- | --- |
|  |  |  |  |  |  |  |  |  |  |  |
|  | **HJ1** | **HJ2** | **YL1** | **YL2** | **JC1** | **JC2** | **AK1** | **AK2** | **QJJ1** | **QJJ2** |
| PH | 6 | 6.5 | 6.7 | 6.2 | 6.8 | 6.4 | 8.13 | 8.06 | 8.47 | 7.8 |
| Cl^-^ (ppm) | 11.7 | 13.6 | 12.3 | 13.6 | 10.4 | 11.6 | 56.1 | 63.7 | 43.2 | 44.2 |
| Ca^2+^(ppm) | 6.4 | 8.7 | 8.9 | 9.2 | 11.2 | 10.7 | 18.8 | 27.4 | 10. 6 | 10.9 |
| Mg^2+^(ppm) | 1.3 | 1 | 2.1 | 2.2 | 2.4 | 3.2 | 4 | 4.9 | 4.4 | 3.3 |
| K^+^(ppm) | 0.5 | 0.7 | 0.75 | 0.78 | 0.8 | 0.9 | 0.2 | 0.2 | 0.1 | 0.1 |
| Na^+^(ppm) | 13 | 10.7 | 15 | 13.3 | 30.9 | 40.7 | 54.9 | 68.4 | 21.6 | 15.4 |
| Fe^2/3+^(ppm) | 10.7 | 11.5 | 17.2 | 18.3 | 16.5 | 13.5 | 8. 7 | 7.9 | 5.2 | 4.3 |
| Mn^2+^(ppm) | 4.3 | 2.1 | 3.3 | 3.1 | 4 | 2.8 | 10.9 | 11 | 6.8 | 7.9 |
| Cu^2+^(ppm) | 0.4 | 1.4 | 0.8 | 0.9 | 1.1 | 1.2 | 0.1 | 0.1 | 0.1 | 0.1 |
| Zn^2+^(ppm) | 0.9 | 1 | 0.5 | 0.4 | 0.7 | 0.9 | 0.1 | 0.1 | 0.1 | 0.1 |
| Salinity (%) | 3 | 4 | 5 | 6 | 0.4 | 0.6 | 11.7 | 10 | 7.8 | 9.4 |
| Total N | 12.6 | 10.4 | 13.5 | 15.1 | 35.9 | 20.4 | 15.6 | 16.5 | 12 | 12.3 |
| Total P | 1.2 | 4.2 | 3.2 | 2.7 | 2.6 | 3.2 | 0.8 | 0.6 | 0.9 | 1 |

Table S2. Geographic distance (kilometers) among 10 different sampling sites

| sample sites | Subtropical monsoon climate | | | | | | Temperate and arid climate | | | |
| --- | --- | --- | --- | --- | --- | --- | --- | --- | --- | --- |
|  | HJ1 | HJ2 | YL1 | YL2 | JC1 | JC2 | AK1 | AK2 | QJJ1 | QJJ2 |
| HJ1 | 0 | 2.4 | 730 | 739 | 565 | 561 | 4433 | 4427 | 3353 | 3264 |
| HJ2 | 2.4 | 0 | 729 | 738 | 569 | 568 | 4435 | 4429 | 3357 | 3267 |
| YL1 | 730 | 729 | 0 | 8 | 640 | 637 | 3980 | 3987 | 2900 | 2893 |
| YL2 | 739 | 738 | 8 | 0 | 650 | 649 | 4000 | 4000 | 2920 | 29000 |
| JC1 | 565 | 569 | 640 | 650 | 0 | 12.7 | 4461 | 4465 | 4144 | 4143 |
| JC2 | 561 | 568 | 637 | 649 | 12.7 | 0 | 4458 | 4460 | 4140 | 4138 |
| AK1 | 4433 | 4435 | 3980 | 4000 | 4461 | 4458 | 0 | 5 | 140 | 130 |
| AK2 | 4427 | 4429 | 3987 | 4000 | 4458 | 4455 | 5 | 0 | 126 | 112 |
| QJJ1 | 3353 | 3357 | 2900 | 2920 | 4144 | 4140 | 140 | 126 | 0 | 3.5 |
| QJJ2 | 3264 | 3267 | 2893 | 2900 | 4143 | 4138 | 130 | 112 | 3.5 | 0 |

**Table S3. Isolation and OTUs designation of *Streptomyces* spp. strains, and their STs.** QJJ and AK indicate the Qijiaojing and Aydingkol sampling sites in Xinjiang Province, respectively; and JC and HJ indicate the Jiangcheng and Heijing sampling sites in Yunnan province, respectively.

| Streptomyces strain | Most closet relative Streptomyces type strain | OTU designation | Isolation site | Genotype STs | Ecotype STs |
| --- | --- | --- | --- | --- | --- |
| WS 032 | *Streptomyces lunaelactis* MM109 ^T^ | *Streptomyces lunaelactis* | JC2 | 21 | 16 |
| WS 034 | *Streptomyces lunaelactis* MM109 ^T^ | *Streptomyces lunaelactis* | JC2 | 21 | 16 |
| WS 043 | *Streptomyces lunaelactis* MM109 ^T^ | *Streptomyces lunaelactis* | HJ1 | 22 | 17 |
| WS 014 | *Streptomyces lunaelactis* MM109 ^T^ | *Streptomyces lunaelactis* | YL2 | 23 | 15 |
| WS 018 | *Streptomyces lunaelactis* MM109 ^T^ | *Streptomyces lunaelactis* | YL2 | 23 | 15 |
| WS 017 | *Streptomyces lunaelactis* MM109 ^T^ | *Streptomyces lunaelactis* | YL2 | 17 | 14 |
| WS 006 | *Streptomyces lunaelactis* MM109 ^T^ | *Streptomyces lunaelactis* | YL1 | 17 | 14 |
| WS 064 | *Streptomyces lunaelactis* MM109 ^T^ | *Streptomyces lunaelactis* | AK1 | 20 | 18 |
| WS 069 | *Streptomyces lunaelactis* MM109 ^T^ | *Streptomyces lunaelactis* | AK1 | 19 | 18 |
| WS 107 | *Streptomyces lunaelactis* MM109 ^T^ | *Streptomyces lunaelactis* | QQJ2 | 18 | 19 |
| WS 095 | *Streptomyces lunaelactis* MM109 ^T^ | *Streptomyces lunaelactis* | QQJ1 | 18 | 19 |
| WS 045 | *Streptomyces griseus* KCTC 9080 ^T^ | *Streptomyces griseus* | HJ1 | 3 | 5 |
| WS 056 | *Streptomyces griseus* KCTC 9080 ^T^ | *Streptomyces griseus* | HJ2 | 4 | 7 |
| WS 058 | *Streptomyces griseus* KCTC 9080 ^T^ | *Streptomyces griseus* | HJ2 | 4 | 7 |
| WS 049 | *Streptomyces griseus* KCTC 9080 ^T^ | *Streptomyces griseus* | HJ1 | 3 | 5 |
| WS 040 | *Streptomyces griseus* KCTC 9080 ^T^ | *Streptomyces griseus* | HJ1 | 3 | 6 |
| WS 073 | *Streptomyces griseus* KCTC 9080 ^T^ | *Streptomyces griseus* | AK1 | 5 | 4 |
| WS 086 | *Streptomyces griseus* KCTC 9080 ^T^ | *Streptomyces griseus* | AK2 | 5 | 3 |
| WS 089 | *Streptomyces griseus* KCTC 9080 ^T^ | *Streptomyces griseus* | AK2 | 5 | 3 |
| WS 079 | *Streptomyces griseus* KCTC 9080 ^T^ | *Streptomyces griseus* | AK1 | 5 | 4 |
| WS 008 | *Streptomyces griseus* KCTC 9080 ^T^ | *Streptomyces griseus* | YL1 | 1 | 1 |
| WS 011 | *Streptomyces griseus* KCTC 9080 ^T^ | *Streptomyces griseus* | YL2 | 1 | 1 |
| WS 027 | *Streptomyces griseus* KCTC 9080 ^T^ | *Streptomyces griseus* | JC1 | 2 | 2 |
| WS 088 | *Streptomyces pratensis* ch 24^T^ | *Streptomyces pratensis* | AK2 | 6 | 8 |
| WS 097 | *Streptomyces pratensis* ch 24^T^ | *Streptomyces pratensis* | QQJ1 | 6 | 8 |
| WS 100 | *Streptomyces pratensis* ch 24^T^ | *Streptomyces pratensis* | QQJ2 | 7 | 8 |
| WS 102 | *Streptomyces pratensis* ch 24^T^ | *Streptomyces pratensis* | QQJ2 | 9 | 8 |
| WS 113 | *Streptomyces pratensis* ch 24^T^ | *Streptomyces pratensis* | QQJ2 | 10 | 8 |
| WS 115 | *Streptomyces pratensis* ch 24^T^ | *Streptomyces pratensis* | QQJ2 | 8 | 8 |
| WS 023 | *Streptomyces pratensis* ch 24^T^ | *Streptomyces pratensis* | JC1 | 16 | 12 |
| WS 024 | *Streptomyces pratensis* ch 24^T^ | *Streptomyces pratensis* | JC1 | 16 | 12 |
| WS 026 | *Streptomyces pratensis* ch 24^T^ | *Streptomyces pratensis* | JC1 | 15 | 13 |
| WS 021 | *Streptomyces pratensis* ch 24^T^ | *Streptomyces pratensis* | JC1 | 15 | 13 |
| WS 033 | *Streptomyces pratensis* ch 24^T^ | *Streptomyces pratensis* | JC2 | 11 | 9 |
| WS 038 | *Streptomyces pratensis* ch 24^T^ | *Streptomyces pratensis* | JC2 | 11 | 9 |
| WS 046 | *Streptomyces pratensis* ch 24^T^ | *Streptomyces pratensis* | HJ1 | 14 | 11 |
| WS 052 | *Streptomyces pratensis* ch 24^T^ | *Streptomyces pratensis* | HJ2 | 13 | 10 |
| WS 041 | *Streptomyces pratensis* ch 24^T^ | *Streptomyces pratensis* | HJ1 | 12 | 10 |

**Table S4. GenBank accession numbers of three house-keeping gene sequences of the studied *Streptomyces* spp.**

| Strain No. | *rpoB* | Strain No. | *recA* | Strain No. | *atpD* |
| --- | --- | --- | --- | --- | --- |
| WS069 | KU902625 | WS079 | KU902663 | WS079 | KU902701 |
| WS064 | KU902626 | WS089 | KU902664 | WS073 | KU902702 |
| WS014 | KU902627 | WS086 | KU902665 | WS086 | KU902703 |
| WS017 | KU902628 | WS056 | KU902666 | WS089 | KU902704 |
| WS018 | KU902629 | WS011 | KU902667 | WS011 | KU902705 |
| WS006 | KU902630 | WS027 | KU902668 | WS008 | KU902706 |
| WS034 | KU902631 | WS008 | KU902669 | WS027 | KU902707 |
| WS043 | KU902632 | WS073 | KU902670 | WS040 | KU902708 |
| WS032 | KU902633 | WS049 | KU902671 | WS049 | KU902709 |
| WS095 | KU902634 | WS040 | KU902672 | WS058 | KU902710 |
| WS107 | KU902635 | WS045 | KU902673 | WS045 | KU902711 |
| WS102 | KU902636 | WS058 | KU902674 | WS056 | KU902712 |
| WS113 | KU902637 | WS100 | KU902675 | WS102 | KU902713 |
| WS115 | KU902638 | WS033 | KU902676 | WS023 | KU902714 |
| WS097 | KU902639 | WS088 | KU902677 | WS033 | KU902715 |
| WS088 | KU902640 | WS097 | KU902678 | WS024 | KU902716 |
| WS100 | KU902641 | WS038 | KU902679 | WS038 | KU902717 |
| WS052 | KU902642 | WS026 | KU902680 | WS088 | KU902718 |
| WS033 | KU902643 | WS102 | KU902681 | WS097 | KU902719 |
| WS038 | KU902644 | WS024 | KU902682 | WS113 | KU902720 |
| WS041 | KU902645 | WS046 | KU902683 | WS100 | KU902721 |
| WS026 | KU902646 | WS113 | KU902684 | WS115 | KU902722 |
| WS046 | KU902647 | WS115 | KU902685 | WS026 | KU902723 |
| WS024 | KU902648 | WS023 | KU902686 | WS041 | KU902724 |
| WS023 | KU902649 | WS021 | KU902687 | WS046 | KU902725 |
| WS021 | KU902650 | WS052 | KU902688 | WS052 | KU902726 |
| WS079 | KU902651 | WS041 | KU902689 | WS021 | KU902727 |
| WS086 | KU902652 | WS032 | KU902690 | WS107 | KU902728 |
| WS011 | KU902653 | WS034 | KU902691 | WS095 | KU902729 |
| WS008 | KU902654 | WS043 | KU902692 | WS017 | KU902730 |
| WS056 | KU902655 | WS018 | KU902693 | WS014 | KU902731 |
| WS045 | KU902656 | WS014 | KU902694 | WS043 | KU902732 |
| WS058 | KU902657 | WS095 | KU902695 | WS018 | KU902733 |
| WS073 | KU902658 | WS107 | KU902696 | WS032 | KU902734 |
| WS040 | KU902659 | WS017 | KU902697 | WS069 | KU902735 |
| WS027 | KU902660 | WS006 | KU902698 | WS064 | KU902736 |
| WS089 | KU902661 | WS069 | KU902699 | WS006 | KU902737 |
| WS049 | KU902662 | WS064 | KU902700 | WS034 | KU902738 |

**Table S5. GenBank accession numbers of four salty-tolerence related gene sequences of the studied *Streptomyces*** spp.

| Strain No. | *ectA* | Strain No. | *ectB* | Strain No. | *ectC* | Strain No. | *ectD* |
| --- | --- | --- | --- | --- | --- | --- | --- |
| WS102 | KU902739 | WS073 | KU902777 | WS115 | KU902814 | WS049 | KU902852 |
| WS033 | KU902740 | WS008 | KU902778 | WS032 | KU902815 | WS045 | KU902853 |
| WS041 | KU902741 | WS045 | KU902779 | WS034 | KU902816 | WS027 | KU902854 |
| WS113 | KU902742 | WS056 | KU902780 | WS006 | KU902817 | WS008 | KU902855 |
| WS088 | KU902743 | WS049 | KU902781 | WS017 | KU902818 | WS079 | KU902856 |
| WS097 | KU902744 | WS089 | KU902782 | WS026 | KU902819 | WS086 | KU902857 |
| WS100 | KU902745 | WS086 | KU902783 | WS102 | KU902820 | WS073 | KU902858 |
| WS038 | KU902746 | WS058 | KU902784 | WS038 | KU902821 | WS058 | KU902859 |
| WS115 | KU902747 | WS027 | KU902785 | WS014 | KU902822 | WS011 | KU902860 |
| WS026 | KU902748 | WS079 | KU902786 | WS018 | KU902823 | WS056 | KU902861 |
| WS021 | KU902749 | WS011 | KU902787 | WS113 | KU902824 | WS040 | KU902862 |
| WS024 | KU902750 | WS026 | KU902788 | WS086 | KU902825 | WS089 | KU902863 |
| WS023 | KU902751 | WS024 | KU902789 | WS089 | KU902826 | WS088 | KU902864 |
| WS052 | KU902752 | WS021 | KU902790 | WS049 | KU902827 | WS115 | KU902865 |
| WS046 | KU902753 | WS023 | KU902791 | WS040 | KU902828 | WS102 | KU902866 |
| WS056 | KU902754 | WS040 | KU902792 | WS045 | KU902829 | WS097 | KU902867 |
| WS040 | KU902755 | WS097 | KU902793 | WS079 | KU902830 | WS021 | KU902868 |
| WS045 | KU902756 | WS100 | KU902794 | WS073 | KU902831 | WS026 | KU902869 |
| WS049 | KU902757 | WS113 | KU902795 | WS056 | KU902832 | WS100 | KU902870 |
| WS058 | KU902758 | WS115 | KU902796 | WS058 | KU902833 | WS046 | KU902871 |
| WS086 | KU902759 | WS102 | KU902797 | WS043 | KU902834 | WS023 | KU902872 |
| WS089 | KU902760 | WS018 | KU902798 | WS046 | KU902835 | WS024 | KU902873 |
| WS073 | KU902761 | WS006 | KU902799 | WS041 | KU902836 | WS113 | KU902874 |
| WS079 | KU902762 | WS017 | KU902800 | WS052 | KU902837 | WS033 | KU902875 |
| WS011 | KU902763 | WS043 | KU902801 | WS023 | KU902838 | WS038 | KU902876 |
| WS008 | KU902764 | WS034 | KU902802 | WS024 | KU902839 | WS041 | KU902877 |
| WS027 | KU902765 | WS014 | KU902803 | WS021 | KU902840 | WS052 | KU902878 |
| WS006 | KU902766 | WS107 | KU902804 | WS095 | KU902841 | WS034 | KU902879 |
| WS095 | KU902767 | WS095 | KU902805 | WS107 | KU902842 | WS032 | KU902880 |
| WS069 | KU902768 | WS032 | KU902806 | WS033 | KU902843 | WS043 | KU902881 |
| WS064 | KU902769 | WS069 | KU902807 | WS100 | KU902844 | WS014 | KU902882 |
| WS018 | KU902770 | WS064 | KU902808 | WS008 | KU902845 | WS018 | KU902883 |
| WS017 | KU902771 | WS033 | KU902809 | WS011 | KU902846 | WS107 | KU902884 |
| WS014 | KU902772 | WS038 | KU902810 | WS027 | KU902847 | WS064 | KU902885 |
| WS107 | KU902773 | WS046 | KU902811 | WS097 | KU902848 | WS017 | KU902886 |
| WS032 | KU902774 | WS052 | KU902812 | WS088 | KU902849 | WS006 | KU902887 |
| WS043 | KU902775 | WS041 | KU902813 | WS064 | KU902850 | WS069 | KU902888 |
| WS034 | KU902776 |  |  | WS069 | KU902851 | WS095 | KU902889 |

**Figure captions**

**Fig. S1. Neighbour-joining phylogenetic trees of concatenated sequences of four house-keeping genes (indicated by A, 16S rRNA, *rpoB*, *recA* and *atpD*) and salty-tolerented related genes (indicated by B, ect*A*-*D*), showing endemism for three *Streptomyces* OTUs.** Bar, 0.05, five nucleotide substitutions per 1000nt; Bootstrap values are shown as percentage of 1000 replicates, and only the bootstrap values above 50% are shown. Solid squares(■), pright triangles (▲), inverse triangles (▼), diamonds (◆) and black circles (●) indicate *Streptomyces* strains from Qijiaojing (QJJ) and Aydingkol (AK) sampling sites of Xinjiang Province and Jiangcheng (JC), Heijing (HJ), Yunlong(YL) sampling sites of Yunnan Province, respectively. The geno- and ecotypes are marked in the clades, and each genotype is supported by high bootstrap value (>80%).

**Fig.S2. Relative expression quantitation of *ectA*-*D* genes at 5 % of NaCl by Q-PCR.** Solid squares(■), pright triangles (▲), inverse triangles (▼), diamonds (◆) and black circles (●) indicate *Streptomyces* strains from Qijiaojing (QJJ) and Aydingkol (AK) sampling sites of Xinjiang Province and Jiangcheng (JC), Heijing (HJ), Yunlong(YL) sampling sites of Yunnan Province, respectively

**Fig.S3. Selection plots of three house-keeping genes and four salty-tolerence related genes.** A-G penals denote *rpoB*, *recA*, *atpD* and *ect A*-*D*. The dN-dS ratios ω for each specific model were used to detect the studied genes undergoing positive selection, ω>1 indicates residues of genes under positive selection, p-value < 0.05.

*Streptomyces griseus* WS 040

*Streptomyces griseus* WS 045

Genotype 3

*Streptomyces griseus* WS 058

*Streptomyces griseus* WS 056

Genotype 4

*Streptomyces griseus* WS 011

*Streptomyces griseus* WS 008

Genotype 1

*Streptomyces griseus* WS 027

*Streptomyces griseus* KCTC 9080^T^

Genotype 2

*Streptomyces griseus* WS 079

*Streptomyces griseus* WS 073

*Streptomyces griseus* WS 089

*Streptomyces griseus* WS 086

Genotype 5

*Streptomyces griseus*

*Streptomyces anulatus* AS 4.1421^T^

*Streptomyces alboviridis* AS 4.1627^T^

*Streptomyces acrimycini* AS 4.1673^T^

*Streptomyces caviscabies* AS 4.1836^T^

*Streptomyces californicus* AS 4.570^T^

*Streptomyces albovinaceus* AS 4.1631^T^

*Streptomyces badius* AS 4.1406^T^

*Streptomyces pratensis* WS 033

*Streptomyces pratensis* WS 038

Genotype 11

Genotype 12

*Streptomyces pratensis* WS 041

Genotype 13

*Streptomyces pratensis* WS 052

*Streptomyces pratensis* HQA015^T^

*Streptomyces pratensis* WS 046

Genotype 14

*Streptomyces pratensis* WS 021

*Streptomyces pratensis* WS 026

Genotype 15

*Streptomyces pratensis* WS 023

*Streptomyces pratensis* WS 024

Genotype 16

Genotype 10

*Streptomyces pratensis* WS 113

Genotype 9

*Streptomyces pratensis* WS 102

Genotype 8

*Streptomyces pratensis* WS 115

Genotype 7

*Streptomyces pratensis* WS 100

*Streptomyces pratensis* WS 097

*Streptomyces pratensis* WS 088

Genotype 6

*Streptomyces atroolivaceus* AS 4.1405^T^

*Streptomyces aureus* AS 4.1833^T^

*Streptomyces lunaelactis* WS 034

*Streptomyces lunaelactis* WS 032

Genotype 21

Genotype 22

*Streptomyces lunaelactis* WS 043

*Streptomyces lunaelactis* MM25^T^

*Streptomyces lunaelactis* WS 017

*Streptomyces lunaelactis* WS 006

Genotype 17

*Streptomyces lunaelactis* WS 018

*Streptomyces lunaelactis* WS 014

Genotype 23

Genotype 20

*Streptomyces lunaelactis* WS 064

Genotype 19

*Streptomyces lunaelactis* WS 069

*Streptomyces lunaelactis* WS 095

*Streptomyces lunaelactis* WS 107

Genotype 18

*Streptomyces bobili* AS 4.1624^T^

*Streptomyces argenteolus* AS 4.1693^T^

*Mycobacterium tuberculosis* H37Rv^T^

100

80

100

100

98

88

82

80

83

100

82

100

100

85

75

57

76

64

68

97

67

100

100

98

58

58

98

94

83

68

79

100

90

87

81

97

94

98

95

83

100

83

90

96

0.05

*Streptomyces griseus WS 0*49

*Streptomyces pratensis*

*Streptomyces lunaelactis*

**Fig. S1.A**

**Fig. S1.B**

**Fig. S2**


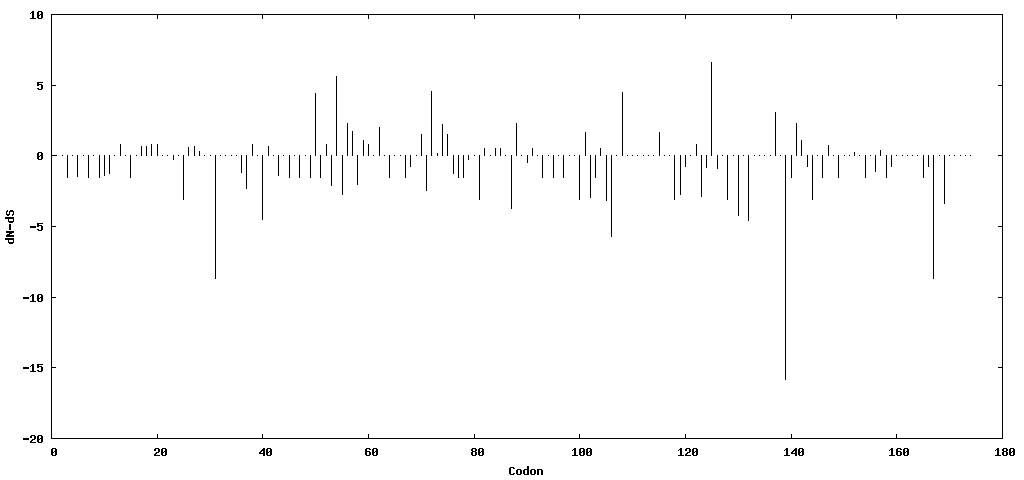


**Fig.S3.A.** Selection plot of *rpoB* gene

**
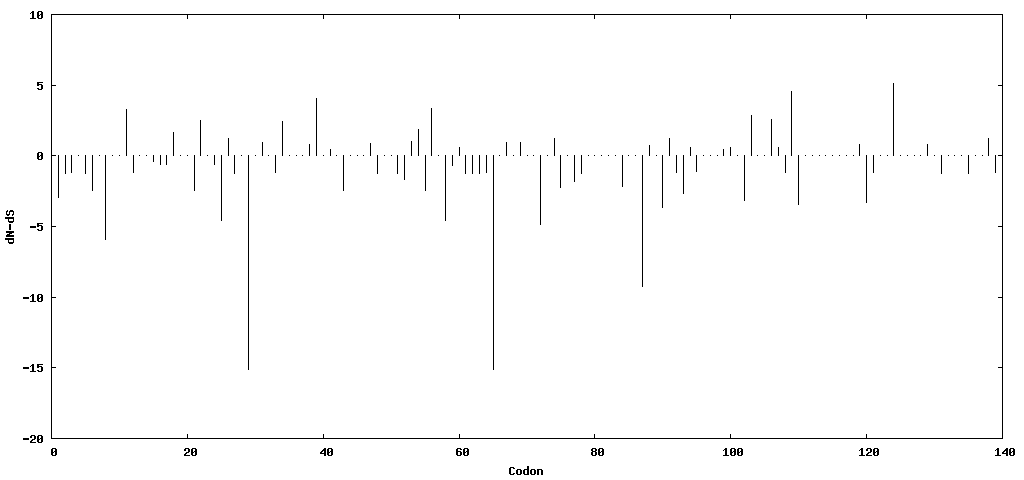
**

**Fig.S3.B.** Selection plot of *recA* gene


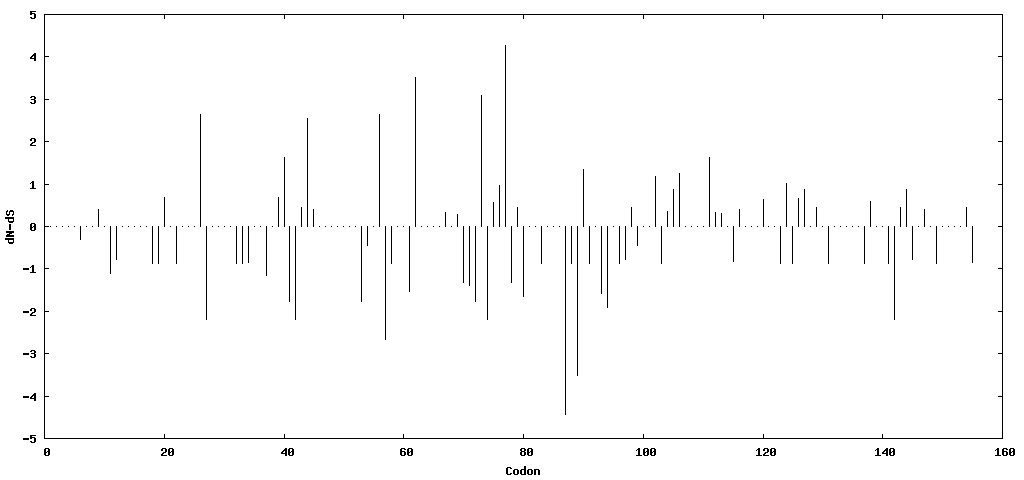


**Fig.S3.C.** Selection plot of *atpD* gene


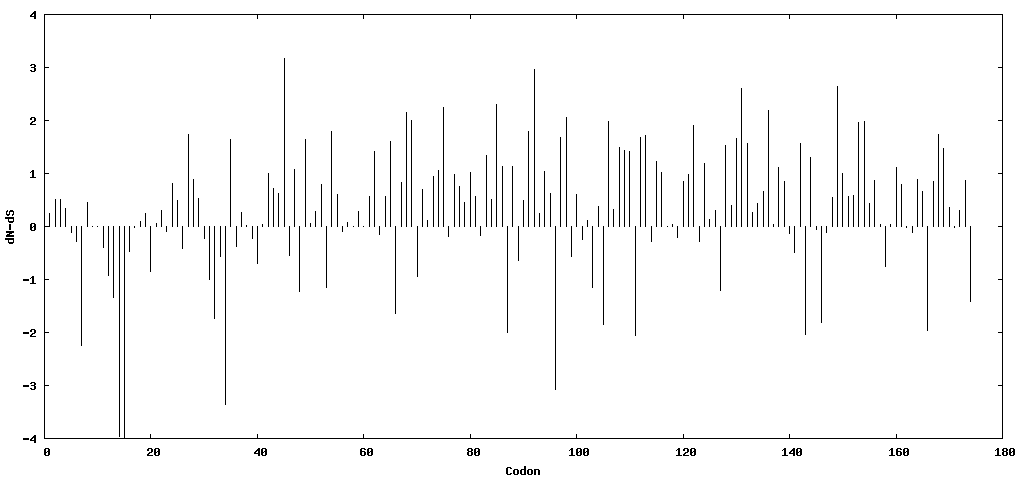


**Fig.S3.D.** Selection plot of *ectA* gene

**
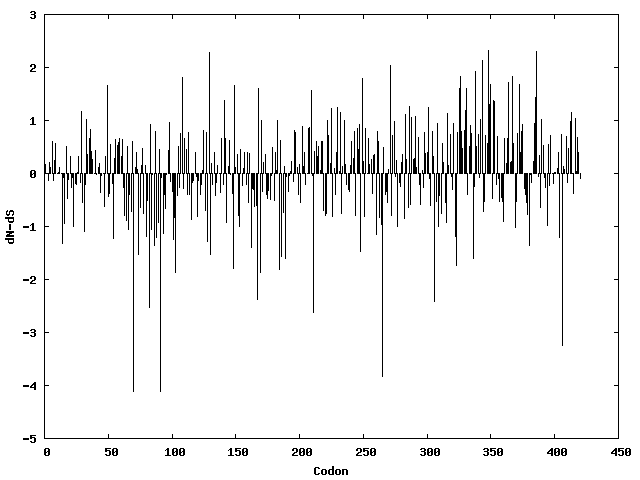
**

**Fig.S3.E.** Selection plot of *ectB* gene


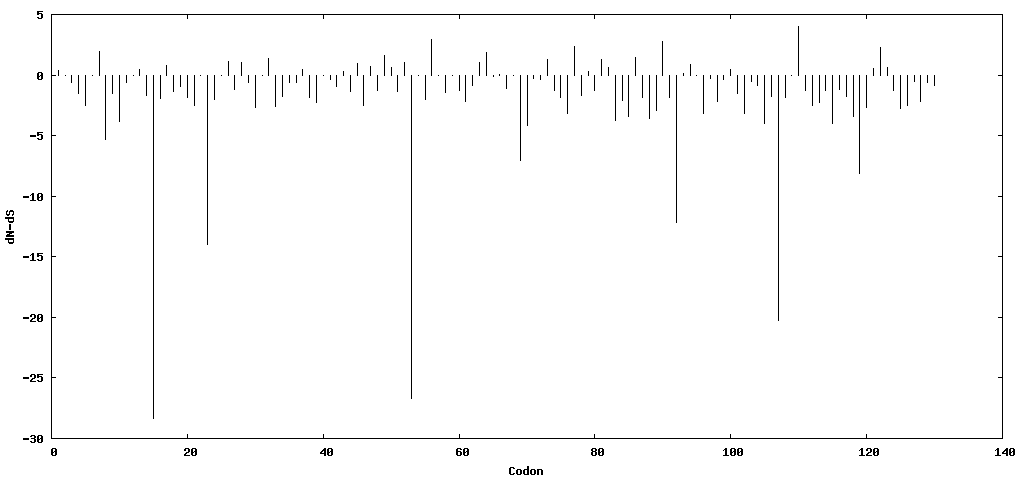


**Fig.S3.F.** Selection plot of *ectC* gene


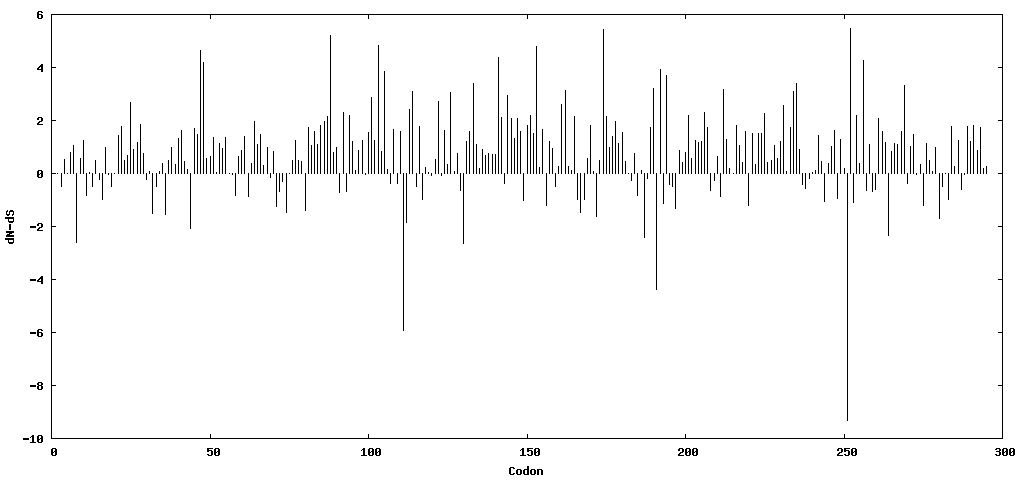


**Fig.S3.G.** Selection plot of *ectD* gene
